# Predictors of early treatment discontinuation and severe anemia in a Brazilian cohort of hepatitis C patients treated with first-generation protease inhibitors

**DOI:** 10.1590/1414-431X20165300

**Published:** 2016-06-23

**Authors:** N. Miotto, L.C. Mendes, L.P. Zanaga, E.S.L. Goncales, M.S.K. Lazarini, M.N. Pedro, F.L. Goncales, R.S.B. Stucchi, A.G. Vigani

**Affiliations:** Divisão de Moléstias Infecciosas, Departamento de Clínica Médica, Faculdade de Ciências Médicas, Universidade Estadual de Campinas, Campinas, SP, Brasil

**Keywords:** Hepatitis C treatment, Protease inhibitor, Anemia, Adverse events, Treatment interruption

## Abstract

The aim of this study was to determine risk factors for adverse events (AE)-related treatment discontinuation and severe anemia among patients with chronic hepatitis C virus (HCV) genotype 1 infection, treated with first-generation protease inhibitor (PI)-based therapy. We included all patients who initiated treatment with PI-based therapy at a Brazilian university hospital between November 2013 and December 2014. We prospectively collected data from medical records using standardized questionnaires and used Epi Info 6.0 for analysis. Severe anemia was defined as hemoglobin ≤8.5 mg/dL. We included 203 patients: 132 treated with telaprevir (TVR) and 71 treated with boceprevir (BOC). AE-related treatment discontinuation rate was 19.2% and anemia was the main reason (38.5%). Risk factors for treatment discontinuation were higher comorbidity index (OR=1.85, CI=1.05-3.25) for BOC, and higher bilirubin count (OR=1.02, CI=1.01-1.04) and lower BMI (OR=0.98, CI=0.96-0.99) for TVR. Severe anemia occurred in 35 (17.2%) patients. Risk factors for this outcome were lower estimated glomerular filtration rate (eGFR; OR=0.95, CI=0.91-0.98) for patients treated with TVR, and higher comorbidity index (OR=2.21, CI=1.04-4.67) and ribavirin dosage (OR=0.84, CI=0.72-0.99) for those treated with BOC. Fifty-five (57.3%) patients treated with TVR and 15 (27.3%) patients treated with BOC achieved sustained virological response (SVR). Among patients who received TVR and interrupted treatment due to AE (n=19), only 26.3% (n=5) achieved SVR (P=0.003). Higher number of comorbidities, lower eGFR and advanced liver disease are associated with severe anemia and early treatment cessation, which may compromise SVR achievement.

## Introduction

Hepatitis C virus (HCV) infection is the leading cause of chronic liver disease and a major public health problem worldwide, affecting 1.1-2% of the global population (1-3). The course of HCV infection and the fibrosis progression rate varies extremely and is influenced by host, viral, and environmental factors (3-6). Patients with chronic HCV infection are at increased risk of developing cirrhosis, hepatic decompensation, and hepatocellular carcinoma (7). Proper and effective antiviral treatment is associated with a reduction in portal hypertension, hepatic decompensation, hepatocellular carcinoma, liver transplantation, and liver-related mortality (3,4).

Since the discovery of the HCV in 1989, treatment options have improved. Interferon alfa (IFN-α) was the first therapeutic option, with sustained virologic response (SVR) rates of 8-21% (8). Afterwards, therapy consisted in IFN-α combined to ribavirin (RBV), which enhanced SVR rates to 40%, and then pegylated IFN-α (PEG-IFN-α) and RBV, with SVR rates of 42-52% (9-11). In 2010, direct antiviral agents (DAA) became available; the first DAA were the protease inhibitors (PI) telaprevir (TVR) and boceprevir (BOC). These drugs are used in combination with PEG-IFN-α and RBV. The SVR among naive patients treated with triple therapy based on TVR or BOC are 75% and 67-68%, respectively (12-16). More recently, new DAA targeting protease, NS5A, and polymerase inhibitors allowed IFN-free effective regimens, with SVR rates above 90% (17,18).

Adverse events (AE) are common in both IFN-α and PEG-IFN-α-based regimens. First-generation PIs increase the rates of certain AE such as anemia, pruritus, rash, gastrointestinal effects, and dysgeusia. Observational cohort studies outside the context of clinical trials demonstrated that AE rates are higher and tolerability of PI-regimens tend to be worse than reported in clinical trials, particularly for patients with comorbidities and cirrhosis (19,20). AE can lead to treatment discontinuation, which may compromise SVR achievement (19-21). Treatment discontinuation rates due to AE in patients treated with RBV associated with IFN-α or PEG-IFN-α were 10 and 12%, respectively (22,23). First-generation PI-based treatment discontinuation rates due to AE vary from 12 to 17% in clinical trials and from 12 to 29% in observational cohorts (19-21). Real-life studies demonstrate that anemia is the most frequent adverse event responsible for PI-based treatment discontinuation (20,21).

Despite of the effectiveness and safety of new DAA, treatments involving these drugs are costly and are an economic burden for many countries. In these settings, first-generation PI-based triple therapy may be a treatment option for certain patients. On the other hand, high rates of serious AE leading to PI discontinuation remain an issue that could compromise treatment outcome. The aim of this study is to determine the risk factors for treatment discontinuation due to AE and severe anemia in a cohort of Brazilian patients treated with TVR- or BOC-based therapy.

## Material and Methods

### Patient enrollment and data collection

We included all patients with HCV genotype 1 chronic infection who started treatment with PEG-IFN-α, RBV, and either TVR or BOC at Hospital de Clínicas, Universidade Estadual de Campinas, from November 2013 through December 2014. Treatment naive patients and patients that previously failed to PEG-IFN-α plus RBV treatment were included. We excluded patients with HIV infection, detectable hepatitis B surface antigen, evidence of hepatic decompensation (ascites, encephalopathy, Child-Pugh B or C), and drug or alcohol abuse. This study was approved by the Ethics Committee of the Universidade Estadual de Campinas, and was conducted in accordance with the Helsinki Declaration.

We collected patient data after every clinical evaluation using standardized questionnaires that included demographic and anthropometric information, medical history, and data on HCV infection such as fibrosis hepatic stage, HCV viral loads, HCV genotype, and previous HCV treatment history. Chronic HCV infection was defined as the presence of HCV antibody (Abott AxSYM Anti-HCV 3.0; Abbott Laboratories, Germany) and detectable serum HCV RNA (Amplicor HCV 3, Roche Diagnostics Systems Inc., USA). Presence of diabetes mellitus was determined according to the American Diabetes Association criteria (24). The severity of comorbidities was estimated using Charlson comorbidity index (CCI) (25). Hepatic histological evaluation was graded and staged according to Metavir scoring system (26). The diagnosis of cirrhosis was made upon histological examination (Metavir stage F4), or a combination of characteristics that included clinical (history of ascites, encephalopathy or variceal bleeding), laboratorial (association of thrombocytopenia, hypoalbuminemia, hyperbilirubinemia, and prolonged prothrombin time), and imaging studies (splenomegaly, portal hypertension, and elastography compatible with Metavir stage F4).

Treatment was proposed to patients following standard practices and guidelines at the outpatient clinic, without influence from the study team. Patients received a combination of TVR or BOC, and PEG-IFN-α 2a (180 mg) or 2b (1.5 µg/kg) and RBV (weight-adjusted dose). We performed a 4-week lead-in with PEG-IFN-α and RBV prior to BOC. Lead-in phase for TVR was optional. 1125 mg of TVR was given twice a day, and 800 mg of BOC was administered 3 times a day, following meals. Changes in PEG-IFN-α and RBV dosages were documented and PI dosage did not change during treatment.

Clinical evaluation and laboratory data tests were performed at baseline and every 4 weeks during treatment or more frequently, if needed. Serum biochemical and hematological analysis included glucose, hemoglobin (Hb), platelets, neutrophils, bilirubin, albumin, creatinine and prothrombin time. Estimated glomerular filtration rate (eGFR) was calculated by Modification of Diet in Renal Disease formula (27). HCV viral loads were determined at baseline and treatment weeks 4, 8, 12, 24 and at follow-up 12 weeks after the end of treatment (SVR-12) using Amplicor HCV 3, Roche Diagnostics Systems Inc. We documented all reported AE and any clinically significant abnormalities throughout the treatment period that led to therapy cessation.

Anemia was defined as mild if Hb was between 10.1 and 12.9 g/dL in males and 10.1 and 11.9 g/dL in females; moderate, if Hb was between 8.6 and 10.0 g/dL, and severe, if Hb ≤8.5 g/dL. Anemia management included RBV dose reduction, use of erythropoiesis-stimulating agents, such as erythropoietin (EPO), and transfusion of packed red blood cell (PRBC). Information about use, dosage and timing of initiation of each strategy was recorded. Anemia management and discontinuation of PI or triple therapy was based on the discretion of the physicians attending each patient.

### Statistical analysis

We performed statistical analysis using Epi Info, version 3.5.4 (CDC, USA). Baseline continuous data were reported as median, and categorical values as frequencies and percentages. Univariate analyses were performed using chi-square, Fisher's, and analysis of variation or Mann-Whitney, as appropriate. A P<0.05 was considered to be statistically significant. Variables with P<0.2 were selected for a backward logistic regression model to evaluate risk factors for severe anemia, treatment discontinuation due to AE and SVR rates. Results are reported as hazard ratios and 95% confidence interval (CI).

## Results

We included 203 patients treated with triple therapy based on TVR or BOC. Table 1 shows patients' characteristics. Among all patients, median age was 52 years, most were male (68.5%) and Caucasian (87.7%). The majority of patients had experienced HCV-treatment (77.3%) and 49.8% had cirrhosis. Liver biopsy was performed in 187 patients; 16 patients had a combination of clinical, laboratory and imaging findings that were compatible with cirrhosis.

**Table:**
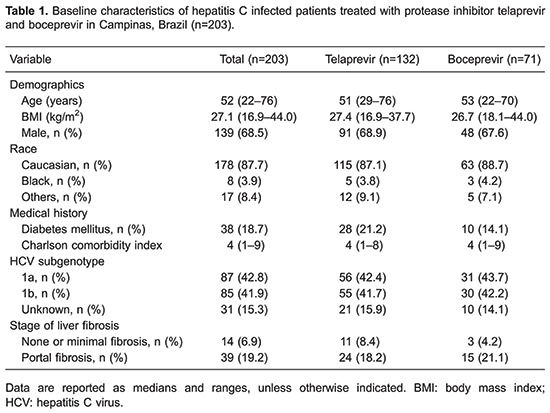

### PI interruption due to AE

Overall, 39 (19.2%) of 203 patients had PI discontinued due to AE, which occurred in 24 (18.1%) of 132 patients treated with TVR and in 15 (21.1%) of 71 treated with BOC. Among patients treated with TVR, anemia was the main reason for PI discontinuation, occurring in 10 (41.6%) of 24 patients, followed by rash in 7 (29.2%), anorectal disorders in 4 (16.6%), cirrhosis decompensation in 1 (4.1%), soft tissue infection in 1 (4.1%), and uncontrollable vomiting in 1 (4.1%). Among patients treated with BOC, anemia was also the main reason that lead to PI discontinuation, occurring in 5 (37.5%) of 15 patients, followed by cirrhosis decompensation in 3 (20%), uncontrollable vomiting in 2 (13.3%), rash in 1 (6.6%), and infection in 1 (6.6%). Three (20%) patients discontinued BOC due to other reasons.

Table 2 shows univariate and multivariate analyses results of factors associated with PI discontinuation due to AE. Concerning patients treated with TVR, univariate analysis demonstrated that higher age, higher CCI, cirrhosis, higher bilirubin count, and lower platelet counts were associated with PI interruption. Multivariate analysis revealed that higher bilirubin count and lower BMI were associated with TVR discontinuation. Among those patients treated with BOC, univariate analysis showed that female gender, lower albumin count, and higher prothrombin international normalized ratio (INR) were associated with PI discontinuation due to AE. Higher CCI was associated with BOC discontinuation in multivariate analysis.

**Table:**
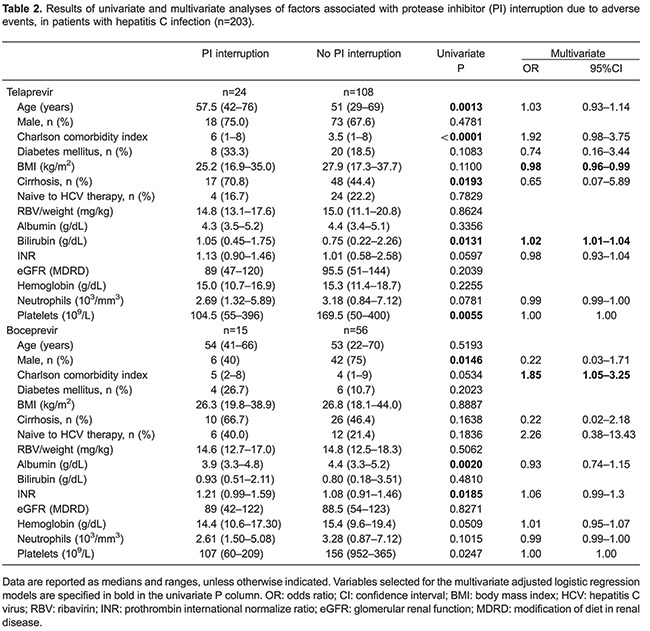

### Anemia

Anemia occurred during treatment in 187 (92.1%) patients and was classified as mild in 87 (42.9%), moderate in 65 (32%), and severe in 35 (17.2%). Table 3 illustrates factors associated with severe anemia. Among patients treated with TVR, older age, female gender, higher CCI, and diabetes mellitus were associated with development of severe anemia. Lower eGFR was associated with development of severe anemia in univariate analysis and multivariate logistic regression. Among patients treated with BOC, female gender, higher BMI, higher CCI, lower baseline albumin, and lower Hb count were associated with development of severe anemia. Higher CCI and higher baseline RBV dosage were associated with development of severe anemia at multivariate logistic regression.

**Table:**
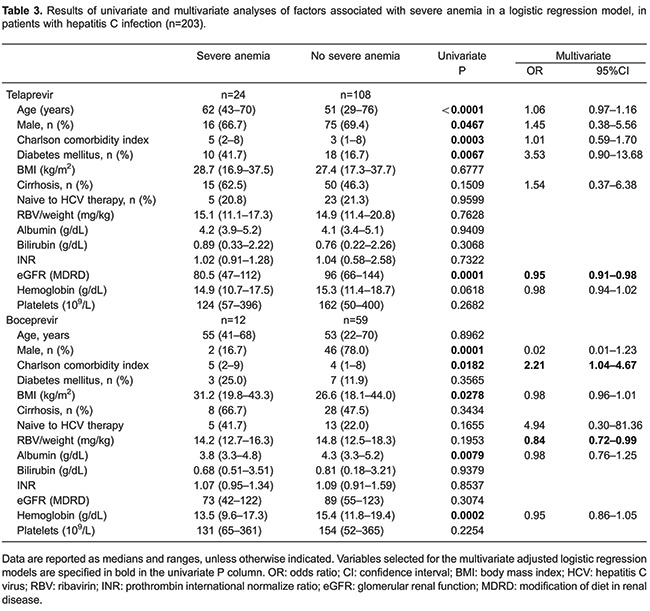

**Figure 1:**
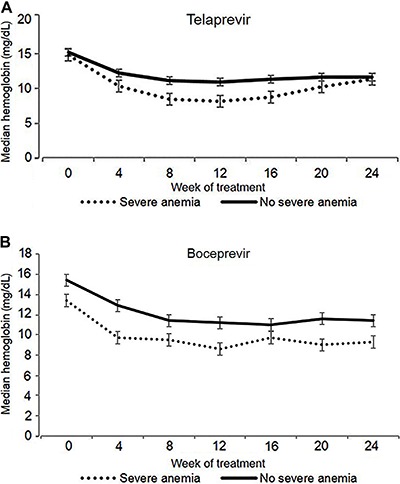
Mean hemoglobin in patients treated for chronic hepatitis C with telaprevir (*A*; n=132) and boceprevir (*B*, n=71). Data are reported as mean±SD.

Figure 1 shows changes in Hb in patients who develop or not severe anemia according to the two PI-based treatments. Among patients treated with TVR, the median time to achieve severe anemia was 8 weeks and the median time to Hb nadir was 12 weeks. In patients treated with BOC, median time to achieve severe anemia and to Hb nadir was 12 weeks. Throughout treatment, Hb remained significantly lower in patients who developed severe anemia for both PI compared to patients who did not develop severe anemia. Strategies used for anemia management were RBV dose reduction [32 patients (15.8%)], EPO [15 (7.4%)], combination of EPO and RBV dose reduction [55 (27%)], EPO and PRBC transfusion [2 (0.9%)], RBV dose reduction and PRBC transfusion [3 (1.5%)] and the three strategies combined [29 (14.2%)]. Median time for first introduction of any anemia treatment was 8 weeks.

### Sustained virological response

During the follow-up period, SVR-12 was available for 151 patients. Among these patients, median age was 51 years; most were male (70.4%), and Caucasian (88.7%). The majority of patients were HCV-treatment-experienced (74.8%) and 49.7% had cirrhosis. Ninety-six (63.6%) received TVR-based therapy and 55 (36.4%) received BOC.

SVR-12 rates were 57.3% (55) in the TVR group and 27.3% (15) in the BOC group. Among patients treated with TVR, SVR rates in 22 previously untreated, 36 relapsers, and 25 non-responders were 81.8, 66.7 and 56.2%, respectively. SVR rates for the subgenotypes were 54.8% (42) for 1a, 56.4% (39) for 1b, and 15 patients were not subgenotyped. Considering fibrosis stage, SVR rate was 100% (9) for minimum fibrosis, 73.3% (15) for portal fibrosis, 55% (20) for bridging fibrosis, and 46% (50) for cirrhosis. In patients treated with BOC, SVR rates in 15 previously untreated patients, 17 relapsers, and 18 non-responders were 46.6, 41.2, and 5.5%, respectively. Regarding the subgenotype, SVR rate was 25% (24) for 1a, 26.1% (17) for 1b, and 21.4% (15) for non-subgenotyped patients. Considering the fibrosis stage, SVR rate was 33.3% (3) for minimum fibrosis, 63.6% (11) for portal fibrosis, 28.6% (14) for bridging fibrosis, and 11.5% (26) for cirrhosis. Table 4 shows factors associated with SVR-12.

**Table:**
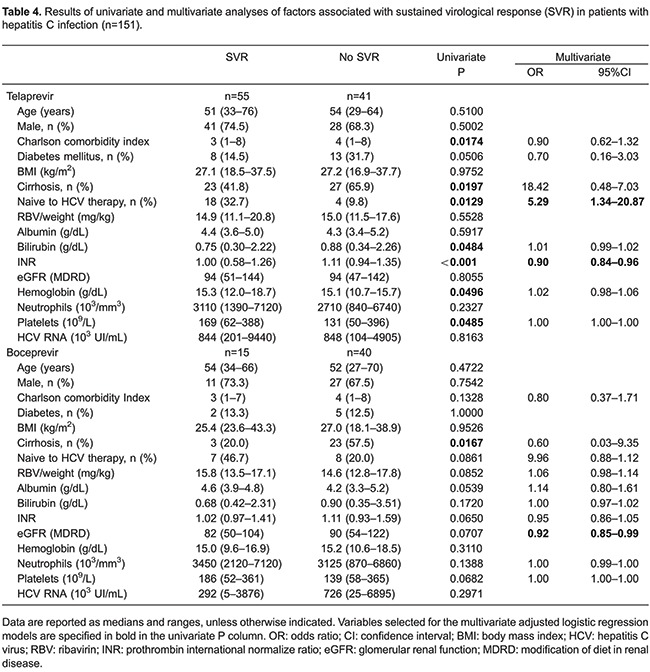

In the TVR group, univariate analysis showed that lower comorbidity index, no previous treatment, absence of cirrhosis, lower bilirubin, lower INR, higher Hb, and higher platelets counts at baseline were associated with higher SVR-12. Multivariate analysis revealed that no pre-treatment and lower INR at baseline were associated with higher SVR-12. Among patients who interrupted treatment due AE (19), only 5 (26.3%) achieved SVR (P=0.003). Among patients treated with BOC, absence of cirrhosis was associated with higher SVR-12 in univariate analysis. Multivariate logistic regression demonstrated that lower eGFR at baseline was associated to higher SVR 12. Only 23% (2) of patients who interrupted therapy due to AE (12) reached SVR (P=0.477).

## Discussion

Our study investigated AE in an observational cohort of HCV infected patients and found that PI interruption due to AE occurred in 19.2% of patients. Treatment interruption was associated with higher comorbidity index, lower BMI, and advanced liver disease. Seventeen percent of patients had severe anemia, which was the main reason for PI discontinuation. Lower eGFR, no prior history for HCV treatment and absence of cirrhosis was associated with higher chances of SVR.

Treatment discontinuation due to AE was higher in our study (19.2%) than in clinical trials for TVR and BOC (10-13 and 8-12%, respectively) which could be explained by the higher proportion of patients with cirrhosis in our study (12-16). However, our AE-related treatment discontinuation rate was similar to the CUPIC cohort (21%), where anemia was also the main reason for PI discontinuation (21). Risk factors for AE-related treatment discontinuation were also consistent with other real-life cohorts, showing that patients with higher number of comorbidities and advanced liver disease are a difficult-to-treat population with higher chances of treatment interruption due to AE (19,21,28,29). Among patients treated with TVR, advanced age and lower BMI was also a risk factor for treatment interruption due to AE (29). Since TVR has fixed dose, patients with lower body mass may have higher drug serum concentration, which may induce more severe AE. Female gender was associated with BOC-based treatment discontinuation due to AE in univariate analysis. However, these data were not corroborated by other cohorts and need further investigation.

Severe anemia rates in our sample was slightly lower (17.2%) than in other reports (22.9-38%) (21,30,31). This could be explained by our definition for severe anemia, which was considered when Hb counts were lower (≤8.5 g/dL) than established by other authors (≤8.9 and ≤10.0 g/dL) (30,31). Predicting factors for the development of severe anemia in multivariate logistic regression were lower eGFR for patients who received TVR, and higher CCI, and baseline RBV dosage for those treated with BOC. We also found that older age and presence of diabetes was associated with severe anemia among patients treated with TVR in univariate analysis, which is comparable to a previous observational cohort (31). Since renal clearance is the major mechanism for clearance of RBV, lower eGFR could lead to higher serum levels of RBV, which is associated with lower Hb levels (32). Presence of multiple comorbidities enhances the chances of multifactorial anemia, possibly contributing to the development of anemia in HCV-infected patients treated with first-generation PI-based regimens (33). These data suggest that patients with renal impairment, older age, and multiple comorbidities should have closer monitoring and early management for anemia to avoid complications such as treatment discontinuation and worsening of clinical status.

Several strategies for the management of anemia in patients receiving triple therapy based on first-generation PI exist. Since PI dosage cannot be reduced due to the risk of resistance development, RBV dose reduction is the main strategy used in the management of anemia in these patients. TVR registration trials prohibited the use of EPO for anemia management, although often experts recommend initiating EPO when Hb levels persist lower than 10 g/dL despite RBV dose reductions. Red blood cell transfusion is an option in the absence of response to other measures or in the presence of clinical symptoms (12,33). The main strategy used in our study was RBV dose reduction associated with EPO, followed by RBV dose reduction alone and the association of the three strategies. Our rate of PRBC transfusion (34%) was slightly lower than reported in previous studies (40-48%), probably because we used it as the last option in order to avoid transfusion-related complications (21,31). Our data show a trend for severe anemia development in patients with Hb ≤10.0 g/dL at week 4 as illustrated in Figure 1. Median interval for initiation of treatment for anemia corresponded to the median time to achieve Hb ≤8.5 g/L (8 weeks), suggesting that early management is important to avoid development of severe anemia.

Overall SVR rate for TVR-treated patients in our study was 57.3%, which is comparable to other observational cohorts (52-60.8%) (34,35). A cohort with 208 treatment naive patients receiving TVR or BOC showed SVR rates of 42% [36)]. Other real-life studies showed SVR rates around 50% (21,28,35). However, the SVR rate for patients treated with BOC in our study (27.3%) was higher than in these studies. The high proportion of patients considered difficult to treat (74.7% of prior non-responders, 75% of advanced fibrosis, and high number of comorbidities), and the relatively small number of patients in this group could explain these results. Two observational cohorts demonstrated that cirrhosis and prior treatment for HCV was associated with lack of SVR (28,35 [Bibr B36]). Likewise, multivariate analysis in our cohort demonstrated that treatment-naive and lower baseline INR patients treated with TVR had higher chances of SVR. Among patients treated with BOC, lower eGFR was associated with SVR. This could be explained by the fact that lower eGFR could decrease drug clearance, exposing patients to higher doses. Lastly, treatment interruption due to AE was associated with lower chances of achieving SVR among TVR-treated patients, highlighting the importance of predicting serious AE in order to allow more timely interventions, potentially reducing the risk of treatment cessation and poor outcome.

Limitations of our study include the population heterogeneity and the relatively small number of patients treated with BOC. We also included patients treated at a single tertiary care outpatient clinic. Since it was an observational study, PI group and strategies used in the management of anemia could not be compared in terms of outcomes. The strength of our study is its focus on patients treated with DAA outside clinical registration trials. To our knowledge, this is the first study to evaluate predicting factors related to severe anemia and treatment discontinuation in Brazil. We demonstrated the relationship between lower eGFR, development of severe anemia and higher chances of SVR.

We believe that it is important to investigate AE and PI interruption rates to evaluate the limitations of first-generation PI-based treatment, and to consider the need for new DAA access. Furthermore, in many countries, new DAA are not extensively available and first-generation PIs are accessible primarily to advanced fibrosis patients. Our findings support that this strategy may expose patients to higher rates of severe anemia, treatment discontinuation and lower SVR rates. In this setting, while new DAA are not universally available, specific cases of previously untreated young patients, with low fibrosis and comorbidity scores could possibly benefit from treatment with triple therapy that are still PEG-IFN-α/RBV based.
